# Improvement of arabinoxylan degradation in *Clostridium saccharobutylicum* DSM 13864^T^ fermentations by heterologous glycoside hydrolase supplementation and expression

**DOI:** 10.1007/s00253-025-13670-4

**Published:** 2025-12-19

**Authors:** Holger Edelmann, Joseph Rebel, Melanie Baudrexl, Wolfgang Liebl, Armin Ehrenreich

**Affiliations:** Chair of Microbiology, Technical University of Munich, Emil-Ramann-Str. 4, 85354 Freising, Germany

**Keywords:** *Clostridium saccharobutylicum*, Arabinoxylan degradation, Cereal bran, Arabinofuranosidase, *Thermoclostridium stercorarium*

## Abstract

**Abstract:**

Solventogenic *Clostridium* species can efficiently produce *n*-butanol and other valuable chemicals via acetone–butanol–ethanol (ABE) fermentation from plant-based feedstocks. For economic and ecological sustainability, cheap and abundant substrates such as lignocellulosic and hemicellulosic residues from agricultural or forestry side streams are preferable. Cereal brans, rich in hemicellulose, represent a promising substrate. However, for direct fermentation of this material, only low product titers are reported. In this study, we characterized the utilization of arabinoxylan, the main polysaccharide component of cereal bran, by the industrial ABE producer *Clostridium saccharobutylicum* DSM 13864^T^ and report inefficient degradation of the substrate. Supplementation with hemicellulolytic enzyme mixtures derived from the thermophilic organism *Thermoclostridium stercorarium* subsp. *stercorarium* DSM 8532^T^ significantly enhanced substrate utilization. The best improvement was achieved by the addition of the arabinofuranosidase Axh43A, which reduced the residual sugar content in the fermentation broth from 48.2 to 17.8%. Analysis of the remaining oligosaccharides after growth on arabinoxylan showed that *C. saccharobutylicum* cannot remove *O*-2 and *O*-3 α-L-arabinofuranosyl groups from double-substituted xyloses, creating a key bottleneck in arabinoxylan degradation that is overcome by Axh43A addition. Plasmid-based expression of Axh43A in *C. saccharobutylicum* DSM 13864^T^ replicated the enzymatic supplementation effects, confirming the enzyme’s role in overcoming this limitation. This underscores the potential of genetic engineering to enhance the valorization of lignocellulosic biomass in biotechnological fermentation processes.

****Key Points**:**

- *C. saccharobutylicum DSM 13864*^T^ *insufficiently utilizes cereal arabinoxylan.*
- *Inability to cleave double-arabinosylated xylose moieties limits degradation.*
- *Heterologous expression of *Axh43A* strongly increases arabinoxylan utilization.*

**Graphical Abstract:**

**Figure:**
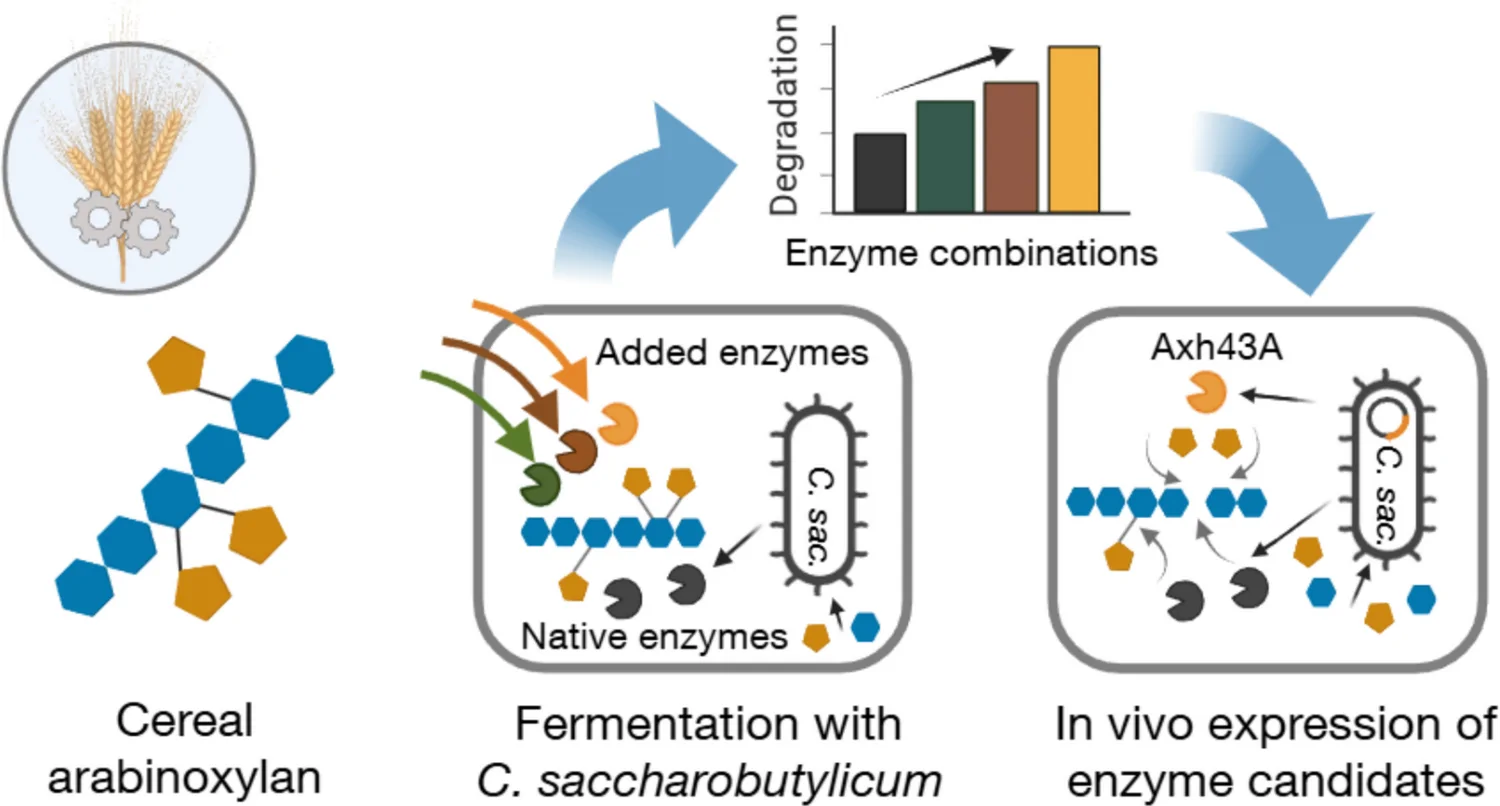

**Supplementary Information:**

The online version contains supplementary material available at 10.1007/s00253-025-13670-4.

## Introduction

Increased awareness of environmental issues and the depletion of fossil resources have led to an increased interest in using renewable feedstock for the sustainable production of fuels and chemicals. Acetone–butanol–ethanol (ABE) fermentation, also called the Weizmann process, can be employed to produce valuable chemicals, such as acetone, ethanol, and butanol from plant-based raw materials, using solventogenic clostridia. The four main species capable of solvent fermentation are *Clostridium acetobutylicum*, *Clostridium beijerinckii*, *Clostridium saccharobutylicum*, and *Clostridium saccharoperbutylacetonicum* (Jones 2024). These species have been typically used in fermentations employing starch-rich biomass, molasses, or sugar hydrolysates as the feedstock (Jones et al. 2023; Dürre 2007).

To make ABE fermentation, once the second largest biotechnological process (Jones and Woods 1986), economically viable again, the use of an abundant and inexpensive substrate is essential. Lignocellulosic biomass (LB), composed of cellulose, hemicellulose, and lignin, meets these criteria (Ponsetto et al. 2024; Lynd et al. 2005), but high ABE-producing solventogenic clostridia have a limited ability to depolymerize cellulose and certain hemicelluloses (Re and Mazzoli 2023; Lee et al. 2009; Al-Shorgani et al. 2012). Thus, chemical, physical, or enzymatical pre-treatment of the plant biomass is required before fermentation, limiting the overall profitability (Olson et al. 2012; Zheng et al. 2022; Gu et al. 2011).

On the other hand, non-solventogenic clostridia, such as *Clostridium thermocellum* (renamed to *Acetivibrio thermocellus)*, *Thermoclostridium stercorarium* (formerly *Clostridium stercorarium*), *Clostridium cellulovorans*, *Ruminiclostridium josi*, and *Clostridium cellulolyticum*, encode numerous enzymes in their genomes that enable them to degrade and utilize lignocellulosic biomass (You et al. 2023). Many of these enzymes have been characterized biochemically (Broeker et al. 2018; Leis et al. 2017; Matsui et al. 2013; Liu et al. 2022; Ravachol et al. 2014; Orita et al. 2017; Wang et al. 2018).

One possible strategy for the efficient conversion of biomass to solvents is to use the natural repertoire of clostridial glycoside hydrolases and transfer them to solventogenic clostridia (Cheng et al. 2019; Mazzoli et al. 2012; Re and Mazzoli 2023; Wen et al. 2020). Due to the phylogenetic relatedness of the various groups of clostridia, their expression and secretion machineries are likely compatible, increasing the chances of successful expression after transfer of such genes. *T. stercorarium* subsp. *stercorarium* DSM8532^T^ is a promising gene donor, as this organism has a diverse set of enzymes for efficient utilization of hemicelluloses (Broeker et al. 2018; Domingues et al. 2022). Especially for hemicellulose hydrolysis, it exceeds and complements the capability of the classical cellulolytic strain *A. thermocellus* (Wang et al. 2022; Froese et al. 2019). *T.* s*tercorarium* DSM8532^T^ does not produce a cellulosome (Zverlov and Schwarz 2008), which may simplify the transfer of its hemicellulolytic enzymes into non-hemicellulolytic strains by avoiding the additional complexity, which showed up during attempts to express cellulosomal enzymes in *C. acetobutylicum* (Chanal et al. 2011; Mingardon et al. 2005, 2011).

Cereal brans are attractive substrates for ABE fermentation. First, they are inexpensive and abundantly available by-products of the milling industry. Second, they contain relatively low amounts of cellulose and lignin, making them less resistant to chemical and biological hydrolysis compared with other lignocellulosic substrates (Apprich et al. 2014). Besides starch (STR) (~ 10–25%), cereal brans also contain a high concentration of the hemicellulose polysaccharide arabinoxylan (AXN) (~ 20–30%) (Chen et al. 2024; Edelmann et al. 2024; Heinze 2005; Knudsen 1997). The content of AXN in milling byproducts is dependent on the cereal species, variety, and also on the milling process (Gebruers et al. 2008). Rye and rye bran possess the highest AXN content (Knudsen 1997). In a previous study, we demonstrated that fermentations of AXN-rich cereal brans using various solventogenic *Clostridium* strains yielded substantially lower butanol titers than fermentations based on starch-rich milling by-products such as wheat middling and rye second flour (Edelmann et al. 2024). This discrepancy was attributed to the insufficient degradation of AXN, which limits the availability of fermentable sugars.

The AXNs from cereals consist of a linear backbone of β-1,4-linked D-xylopyranosyl units which are variably substituted with α-L-arabinofuranosyl groups at *O*-2, *O*-3 (Marcotuli et al. 2016) or both positions. The degree and pattern of substitution depend on the kernel tissue. In wheat, AXN from the aleurone and intermediate layers exhibits an arabinose-to-xylose (A:X) ratio of 0.38, with approximately 5–15% of xyloses being doubly substituted. In contrast, AXN from the outer pericarp is more highly substituted, with an A:X ratio of 1.14 and 34% of xyloses bearing substitutions at both *O-*2 and *O-*3 positions (Chen et al. 2024). Both arabinose and xylose residues may be acetylated at various hydroxyl positions. Additionally, xyloses in the backbone can carry α-1,2- or α-1,4-linked glucuronic or 4-*O-*methylglucuronic acid substituents (Chen et al. 2024). Phenolic acids such as ferulic and *p*-coumaric acids are also present as ester-linked groups at the *O-*5 position of certain arabinose residues (Schendel et al. 2015; Bunzel et al. 2008). These glucuronic acid and phenolic substituents can form covalent ester and ether linkages with lignin, contributing to the cross-linking between polysaccharides and lignin in the plant cell wall (Chateigner-Boutin et al. 2018; Marcotuli et al. 2016; Bunzel et al. 2008; Dervilly 2002).

Efficient depolymerization of AXN requires the synergistic activity of multiple enzymes. The substitution pattern of AXN strongly affects its enzymatic hydrolysis, as side-chain decorations hinder the access of endoxylanases and β-xylosidases to the xylan backbone (Biely et al. 2016, 2013; Pentari et al. 2025). Among these substitutions, arabinose residues are particularly abundant; hence, α-L-arabinofuranosidases play a crucial role in AXN degradation (Pentari et al. 2025; Poria et al. 2020; Xin et al. 2019; Lei et al. 2016). Optimized synthetic enzyme mixtures composed of endoxylanases, β-xylosidases, and arabinofuranosidases have achieved up to 75% and 89% hydrolysis of soluble AXN (Broeker et al. 2018; Sørensen et al. 2007). The inclusion of accessory esterases—capable of removing feruloyl and acetyl substituents—has been shown to significantly enhance the degradation of these recalcitrant substrates (Pereira et al. 2021; Mafa et al. 2021; Biely et al. 2016; Schendel et al. 2015).

Despite detailed knowledge of the structure of cereal AXN (Huang et al. 2024; Chen et al. 2024) and the key enzyme activities required for its hydrolysis (McCleary et al. 2015; Sørensen et al. 2007; Pentari et al. 2025), the AXN degradation capacity of any solventogenic *Clostridium* species remains essentially unexplored. This absence of knowledge is of particular relevance for the biotechnological use of milling by-products like cereal brans, where efficient processing of AXN remains a significant bottleneck. With the current study, we fill this gap by investigating and enhancing wheat AXN utilization in *C. saccharobutylicum* DSM 13864ᵀ by supplementing fermentations with tailored combinations of AXN-degrading enzymes from *T. stercorarium* DSM 8532ᵀ. AXN conversion is evaluated through detailed compositional and oligosaccharide analyses. In addition, to establish a recombinant strategy to supply the solventogenic host with enzyme activities recognized as lacking but necessary for efficient AXN utilization, the heterologous expression of selected enzyme candidates in the host strain was explored. *C. saccharobutylicum* was chosen for this work due to its reported genetic stability, elevated solvent titers, and high fermentation rate (Jones et al. 2023).

## Materials and methods

### Bacterial strains and culture conditions

All bacterial strains used in this study are listed in Table 1. *Escherichia coli* strains were cultivated aerobically at 37 °C with agitation at 180 rpm. Lysogeny Broth (LB) medium was used for growth and, when required, solidified with 1.5% (w/v) agar. Antibiotics were supplemented as appropriate: chloramphenicol (25 mg/L), tetracycline (10 mg/L), and kanamycin (50 mg/L). *E. coli* DH10β was employed for plasmid construction and cloning procedures, while *E. coli* BL21 Star™ (DE3) served as the host strain for protein expression. *C. saccharobutylicum* RD2-A was cultured anaerobically at 37 °C in liquid or solid clostridial growth medium (CGM), supplemented with thiamphenicol (15 mg/L) when required. For spore formation, cultures were first grown in liquid CGM to the exponential phase and subsequently plated onto CGM-agar plates.

**Table 1:** List of bacterial strains and plasmids used in this work

Name	Description	Source
Bacterial strains		
*Clostridium saccharobutylicum* RD2-A	DSM 13864 with ΔCLSA_C29340, ΔCLSA_C04420 similar to Ch1 but recreated (Huang et al. 2018)	Holger Edelmann (unpublished)
*Escherichia coli* BL21 Star (DE3)	F^*−*^* ompT hsdS B (rB*^*−*^* mB*^*−*^*) gal dcm rne131* (DE3)	New England Biolabs (Frankfurt am Main, Germany)
*Escherichia coli* DH10β	F^–^ *mcrA* Δ(*mrr-hsdRMS mcrBC*) φ80*lacZ*ΔM15 Δ*lacX74 recA1 endA1 araD139 Δ(ara-leu)7697 galU galK* λ^–^ *rpsL(StrR) nupG*	New England Biolabs (Frankfurt am Main, Germany)
*Escherichia coli* CA434	R702^+^, F^*−*^* mcrB mrr hsdS20(rB*^*−*^* mB*^*−*^*) recA13 leuB6 ara-14 proA2 lacY1 galK2 xyl-5 mtl-1*	Des Purdy et al. (2002)
Plasmids		
pET-24c(+)	f1 origin, *kanR2*, *lacI*, T7 promoter, *lacO*, sequence for His_*6*_-tag, ColE1 origin	Novagen, Merck (Darmstadt, Germany)
pET-24c(+)-*xynD*	pET-24c(+) with *axh43A* (Cst_c07100), sequence for His_6_-tag was C-terminal fused	Broeker et al. (2018)
pET-24c(+)-*xynB*	pET-24c(+) with *xyn10B* (Cst_c07090), sequence for His_6_-tag was C-terminal fused	Broeker et al. (2018)
pET-24c(+)-*xynA*	pET-24c(+) with */xyn11A* (Cst_c19320), sequence for His_6_-tag was C-terminal fused	Broeker et al. (2018)
pET-24c(+)-*bxl3B*	pET-24c(+) with *bxl3B* (Cst_c19320), sequence for His_6_-tag was C-terminal fused	Broeker et al. (2018)
pMTL83123	*catP*, pCB102 origin, p15A origin, *traJ*, o*riT*, P*fdx* + MCS	Heap et al. (2009)
pJR1	pMTL83123 with *axh43A* (Cst_c07100) cloned into *lacZ*	This work
pJR2	pMTL83123 with *axh43A1* (Cst_c07100 with ΔN759 – E1221, 3’-truncated version of *axh43A*)	This work

### Medium and substrates

CGM medium was prepared as described previously (Wiesenborn et al. 1988) with 2% (w/v) glucose added. CGMr6 was similar to CGM but was supplemented with 200 mM MES buffer (pH 6.0) and contained only 1.5 g/L yeast extract and 1.0 g/L casein amino acids. For fermentation, one or combinations of the carbon sources soluble wheat AXN, soluble STR, or D-glucose were added at a concentration of 0.25% (w/v) each.

Soluble STR was purchased from Merck (Product-Nr. 1.101252; Darmstadt, Germany). Soluble wheat AXN (Product Nr. P-WAXYRS), xylooligosaccharides (XOS), and arabinoxylan oligosaccharides (AXOS) standards were ordered from Megazyme Ltd (Bray, Ireland). P-WAXYRS was extracted by alkali treatment, which should remove acetyl and feruloyl ester bonds, but this was not explicitly analyzed (communication from Megazyme Ltd., Bray, Ireland).

### Enzyme production in *E. coli* and protein purification

For enzyme expression in *E. coli*, the corresponding open reading frame (ORF) was cloned without the sequence encoding the N-terminal signal peptide (predicted by SignalP 4.1 server, default cutoff: 0.3, https://services.healthtech.dtu.dk/services/SignalP-4.1/) but with the addition of a sequence for a C-terminal His_6_-tag (Broeker et al. 2018) in the pET-24c(+) vector from Novagen (Merck, Darmstadt, Germany) (see Table 1). The enzyme was produced in *E. coli* BL21 Star™ in ZYP-5052 auto-induction medium and purified with immobilized metal affinity chromatography (IMAC) as described before (Mechelke et al. 2017). Upon harvesting and disintegration of the cells, the crude extract was heated at 50 °C for 15 min to denature *E. coli* proteins, followed by centrifugation and analysis by sodium dodecyl sulfate–polyacrylamide gel electrophoresis (SDS-PAGE). Representative SDS-PAGE results from the process are provided in the supplementary material (see Supplemental Fig. S1–S4). The enzyme activity was tested by analysis of reducing ends liberated from polysaccharide substrates using the dinitrosalicylic acid (DNSA) assay (Wood and Bhat 1988). The reaction was conducted in a total volume of 150 µL with 0.5% (w/v) wheat AXN with 10 mM CaCl_2_, 100 mM 3-(*N*-morpholino)propanesulfonic acid (MOPS), and 50 mM NaCl at pH 6.0 and incubated for 30 min at 60 °C. Fifty microliters of supernatant after centrifugation was mixed with 75 µL DNSA reagent incubated at 95 °C for exactly 5 min and cooled down on ice. The change in OD at 540 nm was measured in a flat-bottom 96-well plate. To determine the released reducing ends, xylose standards from 0.2 up to 1.4 mg/mL were included on each 96 well plate to generate a calibration curve.

### Cloning

For heterologous expression of the glycoside hydrolase Axh43A in *C. saccharobutylicum*, the shuttle vector pMTL83123 was used. In previous experiments, the low–copy plasmid pMTL83123 with p15A origin of replication appeared to exhibit greater stability in *E. coli* during cloning of constitutively expressed glycosidases than the high–copy plasmid pMTL83153, although this was not quantitatively measured. Literature reports suggest a ∼30-fold disparity in copy number between these two plasmids (∼20 versus ∼600 copies) (Heap et al. 2009; Chambers et al. 1988; Chang and Cohen 1978). Two plasmids were constructed by inserting either the full-length *axh43A* gene (for pJR1) or a truncated variant (for pJR2) into the vector pMTL83123, placing each gene under control of the *fdx* promoter from *Clostridium sporogenes* NCIMB 10696 (Heap et al. 2009). Both constructs retained the native *axh43A* signal peptide-encoding sequence but omitted the C-terminal His_6_-tag (see Supplemental Fig. S5). For the assembly of pJR1 and pJR2, linear DNA fragments corresponding to each *axh43A* variant, containing 30-bp homologous overlaps to the vector backbone, were generated by PCR fusion using Q5 High-Fidelity DNA Polymerase (New England Biolabs GmbH, Frankfurt am Main, Germany). The fragment carrying the full-length *axh43A* ORF was amplified with the primer pair Clst_0676_full_f and Clst_0676_full_r, whereas the truncated version was amplified with primers Clst_0676_full_f and Clst_0676_split_r (see Table 2 and Supplemental Fig. S6). Genomic DNA from *T. stercorarium* DSM 8532^T^ was used as template. Plasmid pMTL83123 was linearized by restriction digestion with *Nde*I and *Xho*I (New England Biolabs GmbH, Frankfurt on the Main, Germany). Assembly of the expression plasmid was carried out using NEBuilder HiFi DNA Assembly Master Mix (New England Biolabs GmbH, Frankfurt on the Main, Germany). Colony PCR using the primer pair pCB102-out-rv and M13_fw, followed by restriction analysis (see Supplemental Fig. S7) was used to verify recombinant plasmids. Additionally, Sanger sequencing of the ORF region in the plasmid was performed with primers Clst_0676_full_f, CLst0676_Seq1, CLst0676_Seq2, and CLst0676_Seq3.

**Table 2:** List of DNA oligonucleotides used as primers

Name	Sequence (5′- > 3′)
Clst_0676_full_f	TTGTGTAATTTTTAAGGAGGTGTGTTACATATGAATACATTTTCCCGAAGAAATC
Clst_0676_full_r	GTGCCAAGCTTGCATGTCTGCAGGCCTCGATCATTCGCTGAAATACCAGTAATC
Clst_0676_split_r	GTGCCAAGCTTGCATGTCTGCAGGCCTCGATCACGGCGGGACTTTCCCTAC
CLst0676_Seq1	GAAGGTTCACACGCATAC
CLst0676_Seq2	TGGTGGATAAAGGCGTTG
CLst0676_Seq3	CACGTACGGAAGGATAAAC
pCB102-out-rv	TCTATTCAGCACTGTTATGCC
M13_fw	CAGGAAACAGCTATGACC

### Conjugation of plasmids into *C. saccharobutylicum*

Introduction of plasmids into *C. saccharobutylicum* was achieved via triparental conjugation. A protocol from Lesiak et al. (2014) was adapted by using CGM medium with 2% glucose and harvesting the cells for mating at an OD_600_ of 0.4. *E. coli* CA434, which harbored the self-mobilizable IncPβ conjugative plasmid pR702, served as the conjugation helper strain. When supplied in trans, pR702 enabled mobilization of *oriT-*containing shuttle vectors and their subsequent transfer into the *Clostridium* recipient strain (Des Purdy et al. 2002).

### Fermentation conditions

Pre-pre-cultures were prepared by inoculation of 5 mL of CGM containing 2% glucose with a fresh colony each of a *C. saccharobutylicum* strain and incubated for 24 h at 37 °C. Thereafter, pre-cultures with CGMr6 medium were inoculated with 0.1 volumes of the grown pre-pre-cultures and incubated 14 h at 37 °C. Cells from pre-cultures were washed once with 1 × phosphate-buffered saline (PBS), and the 5 mL main culture in CGMr6 with 0.25% (w/v) of each substrate was inoculated to an OD_600_ of 0.25–0.4 in Hungate-type tubes. After fermentation for 48 h at 37 °C, samples were taken with a syringe. After sedimentation of the cells by centrifugation, the supernatant was stored at −20 °C for further carbohydrate analysis.

### Acid hydrolysis

Acidic hydrolysis of soluble polysaccharides was done with a modified version of the protocol from National Renewable Energy Laboratory (NREL) (Sluiter et al. 2012). To account for sugar degradation during the thermal process, a sugar recovery solution (SRS) was prepared, which contained each monosaccharide at a concentration of 1 mg/mL. The sample supernatant and in parallel the SRS standard were mixed in a 1:1 ratio with 8.11% (w/v) H₂SO_4_ in screw-cap tubes and heated in an autoclave at 121 °C for 60 min. Monosaccharide analysis was conducted using high-performance anion-exchange chromatography with pulsed amperometric detection (HPAEC-PAD), and the samples were diluted 250-fold before analysis. L-rhamnose served as the internal standard and was introduced in the first dilution step. One part of the sample was mixed with nine parts of an L-rhamnose solution (138.9 mg/L) in the first step; then, subsequent dilutions were performed using pure deionized water (ddH_2_O). The resulting values were corrected to account for thermal degradation of monosaccharides with the SRS_Sugar_ coefficient. SRS_Sugar_ was calculated for each sugar individually by dividing the sugar concentration of the non-autoclaved SRS solution by the concentration of the autoclaved solution. Additionally, the mass increase by H_2_O addition during hydrolysis had to be corrected by dividing by the hydrolysis coefficients of hexoses (HydCor_Hexose_ = 1.10) or pentoses (HydCor_Pentose_ = 1.12).

### HPAEC-PAD measurements

The ICS-6000 system from Thermo Fischer Scientific GmbH (Dreieich, Germany) was used for sugar analysis. The chromatography system was equipped with PEEK tubing, a GM4 gradient mixer (2 mm), a CarboPacTM PA1 column (2 × 200 mm), and a PA1 precolumn (2 × 50 mm). Runs were performed at a column and detector temperature of 30 °C and a flow rate of 0.3 ml/min. Ten-microliter samples were injected. Carbohydrate detection was performed using pulsed amperometric detection (PAD). A conventional gold block electrode with a one mil gasket and a AgCl-pH reference electrode were used with a data collection rate of 2 Hz and a “Gold Carbo, Quad” waveform. Calibration was performed in the range of 0.110 mg/L of each sugar with 5 mg/L rhamnose as internal standard.

The three following eluents were prepared fresh and carbonate-free before each measurement, using sodium acetate from Carl Roth GmbH (Karlsruhe, Germany, PO number 6773.2) and carbonate-free 50% (w/v) NaOH solution from VWR International (Darmstadt, Germany, PO number 87938.290). Eluent A contained 65 mM NaOH, Eluent B contained 65 mM NaOH and 1 M NaOAc, and Eluent C contained only ddH_2_O.

Monosaccharides were separated at 13.5 mM NaOH for 20 min. Then, the column was washed for 5 min with 650 mM NaOAc/65 mM NaOH, and 10 min with 65 mM NaOH. Finally, the column was re-equilibrated for 10 min with 13.5 mM NaOH.

For the separation of oligosaccharides, a linear gradient from 7.5 to 100 mM NaOAc in 67.5 min was used with constant 65 mM NaOH. In a washing step, the concentration of NaOAc was increased for 4 min to 650 mM while leaving the NaOH concentration constant at 65 mM. Subsequent re-equilibration was performed with 65 mM NaOH for 13 min after each run. This method was adapted from Mechelke et al. (2017).

## Results

### Degradation of AXN and STR with *C. saccharobutylicum*

First, a fermentation model was established using AXN and STR, the major polysaccharides from cereal bran. We used modified CGM because *C. saccharobutylicum* DSM 13864^T^ does not grow well on a pure minimal medium. To reduce the sugar content contributed by yeast extract, 70% of the yeast extract was replaced with casein-derived amino acids. Fermentations were conducted with either soluble wheat AXN, STR, glucose, or a mixture of these carbohydrates. Sugars in the supernatant were analyzed with HPAEC-PAD. Smaller oligosaccharides were analyzed directly from the supernatant. Polysaccharides were subjected to acidic hydrolysis prior to their analysis as monosaccharides.

**Fig. 1:**
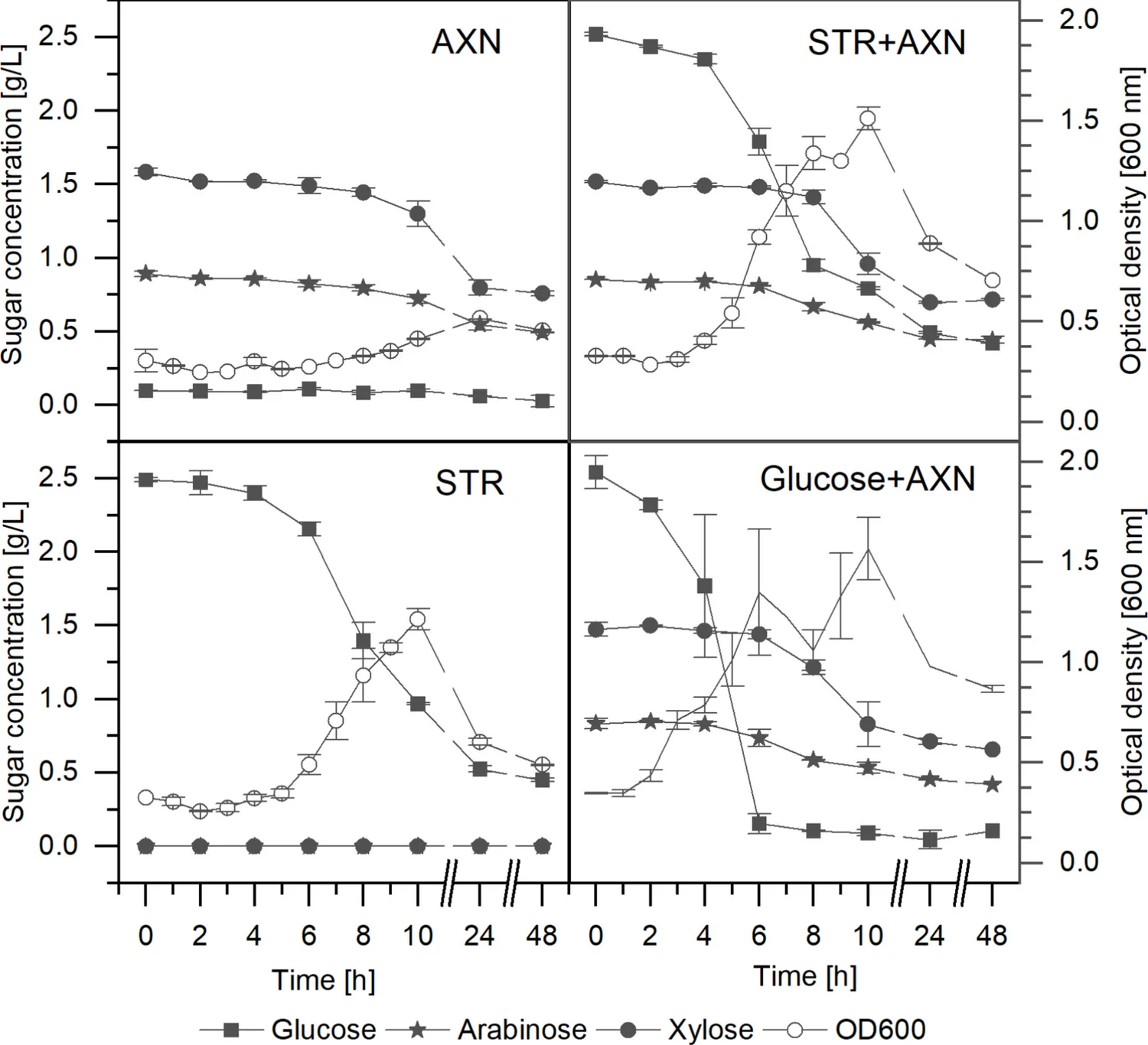
Sugar consumption by *C. saccharobutylicum* RD2-A during growth for 48 h in CGMr6 medium. Fermentations contained the following substrates at concentrations of 0.25% (w/v) each: top left, AXN; top right, STR + AXN; bottom left, STR; and bottom right, glucose + AXN. Samples were collected hourly up to 10 h and subsequently at 24 and 48 h. Glucose was measured directly from the supernatant via HPAEC-PAD analysis while the decreases in STR and AXN contents are shown as monosaccharide concentrations measured using HPAEC-PAD after acid hydrolysis of the polysaccharides. Error bars depict the standard deviation of two biological replicates

As can be seen in Fig. 1, STR and AXN are utilized by *C. saccharobutylicum*. Growth on AXN led to a lower increase in the optical density compared to STR and glucose. Glucose and to a lesser extent also STR induce carbon catabolite repression (CCR) and were utilized faster (Fig. 1). However, when the medium was depleted of these two carbohydrates (after 6–8 h), the strain started to degrade AXN. Depletion of glucose led to a rapid and strong drop in optical density with a subsequent increase during growth on AXN. In all conditions, glucose was the only substrate that was completely utilized. In mixed or single substrate conditions after 48 h, 48.7 ± 1.5% residual AXN and 13.5 ± 0.7% residual STR could be measured.

### Enhanced AXN breakdown and utilization by addition of *T. stercorarium* enzymes

**Fig. 2:**
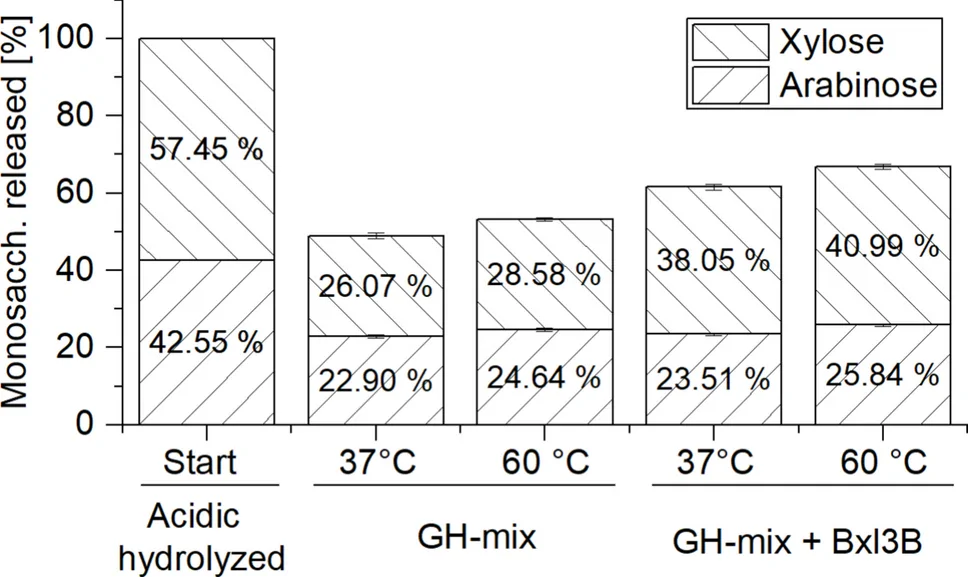
Degradation of 0.25% (w/v) soluble wheat AXN in CGMr6 medium with combinations of AXN-degrading enzymes. Following sterile addition of the GH-enzyme mix––comprising Axh43A, Xyn11A, and Xyn10B—the reactions were incubated for approx. 14 h at either 37 °C or 60 °C. After the withdrawal of samples for sugar analysis, Bxl3A was added to the reaction, and the incubation was continued for an additional 4 h at the same temperature. For the quantification of total carbohydrate content, samples were pretreated by acidic hydrolysis, while enzymatically released sugars could be directly measured with HPAEC-PAD. Final enzyme concentrations were as follows: Axh43A (1.5 μg mL⁻^1^), Xyn11A (5 μg mL⁻^1^), Xyn10B (5 μg mL⁻^1^), and Bxl3A (4 μg mL⁻^1^). Error bars represent the standard deviation of three independent biological replicates

In a previous study, Broeker (2019) identified key enzymes of the hemicellulolytic *T. stercorarium* DSM 8532^T^ that could efficiently hydrolyze wheat AXN. To confirm this effect in our experimental setting, four selected enzymes were heterologously expressed in *E. coli* and purified with IMAC (see “Materials and methods”). Axh43A (1.5 μg/mL), Xyn11A (5 μg/mL), and Xyn10B (5 μg/mL) were added to CGMr6 with 0.25% soluble wheat AXN and incubated overnight at 37 °C and at 60 °C. The latter is the proposed optimal temperature of Axh43A. On the next day, the less stable Bxl3B (4 μg/mL) was added and incubated for an additional 4 h at the same temperature. The total amount of monosaccharides at the starting point was measured after acidic hydrolysis as described in the “Materials and methods” section. After the overnight incubation of pure wheat AXN with the enzyme mix, 48.98 ± 1.53% (37 °C) and 53.22 ± 1.08% (60 °C) of the polymer was released in the form of monosaccharides (Fig. 2). Further addition of the β-xylosidase Bxl3A improved degradation to 61.56 ± 1.05% (37 °C) and 66.83 ± 1.14% (60 °C), which was mostly by releasing additional xylose. Thus, the potential of this four-enzyme cocktail to degrade AXN was confirmed in CGMr6 medium and at 37 °C, which are necessary requirements for fermentation with *C. saccharobutylicum*.

**Fig. 3:**
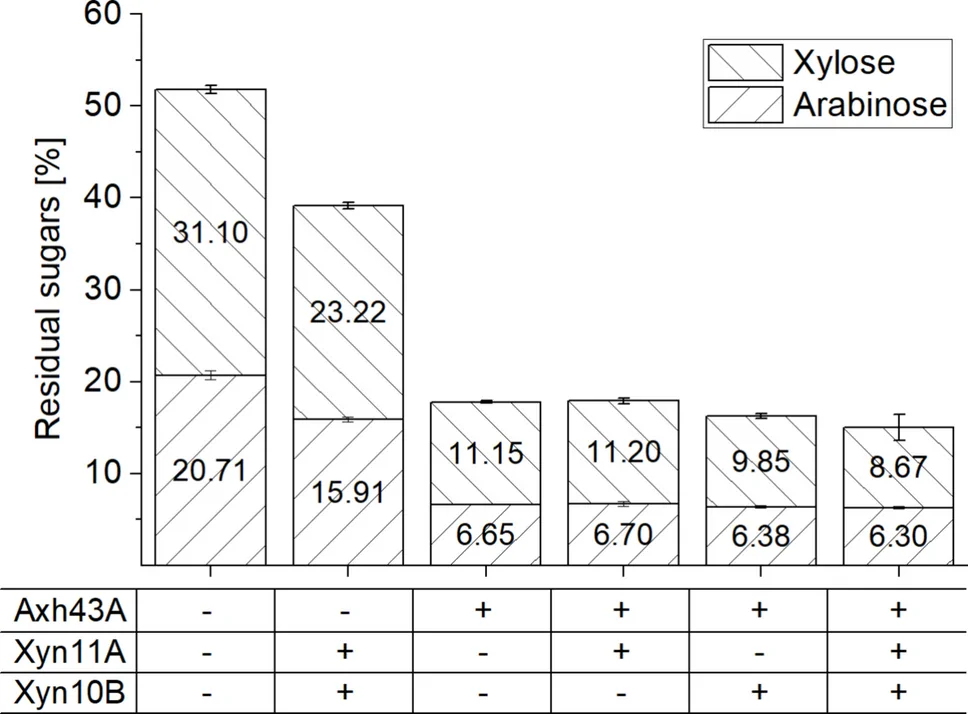
The effect of heterologous enzyme addition to fermentation of 0.25% (w/v) soluble wheat AXN by C*. saccharobutylicum* RD2-A in CGMr6 medium. Enzymes were added individually, in pairs, or as a combined glycoside hydrolases mix (GH-Mix) as indicated in the table. Immediately after the enzymes were added, the medium was inoculated and incubated at 37 °C for 48 h. Final enzyme concentrations were as follows: Axh43A (1.5 μg/mL), Xyn11A (5 μg/mL), and Xyn10B (5 μg/mL). Error bars depict standard deviations of three biological replicates

Upon confirming the strong in vitro activity of the enzyme mixtures on AXN in CGMr6 medium, we continued to assess the biotechnological benefit of the enzyme mix in direct fermentation of AXN with *C. saccharobutylicum*. Therefore, different combinations of the enzymes were added to the medium shortly before inoculation, and the cultures were incubated for 48 h at 37 °C. In accordance with the previous growth experiments on AXN without enzyme supplementation (see above, Fig. 1), 51.8 ± 0.9% residual sugar was measured after fermentation (Fig. 3). The addition of the endo-xylanases Xyn10B and Xyn11A, or of arabinofuranosidase Axh43A, all had a beneficial effect on the degree of AXN utilization. A mixture of Xyn11A and Xyn10B improved AXN degradation and utilization by about 26.3 ± 0.5%, resulting in 39.1 ± 0.6% residual sugar after fermentation. Combinations of each of the endo-xylanases with Axh43A and, interestingly, also Axh43A alone could support degradation to a range of 17.9 ± 0.5–16.2 ± 0.4% residual sugar. Noteworthy, supplementation of the fermentation with all three enzymes (GH-Mix, 15.0 ± 1.5% residual sugar content) had almost the same effect as addition of Axh43A alone (Fig. 3).

### Oligosaccharide analysis of culture supernatant

**Fig. 4:**
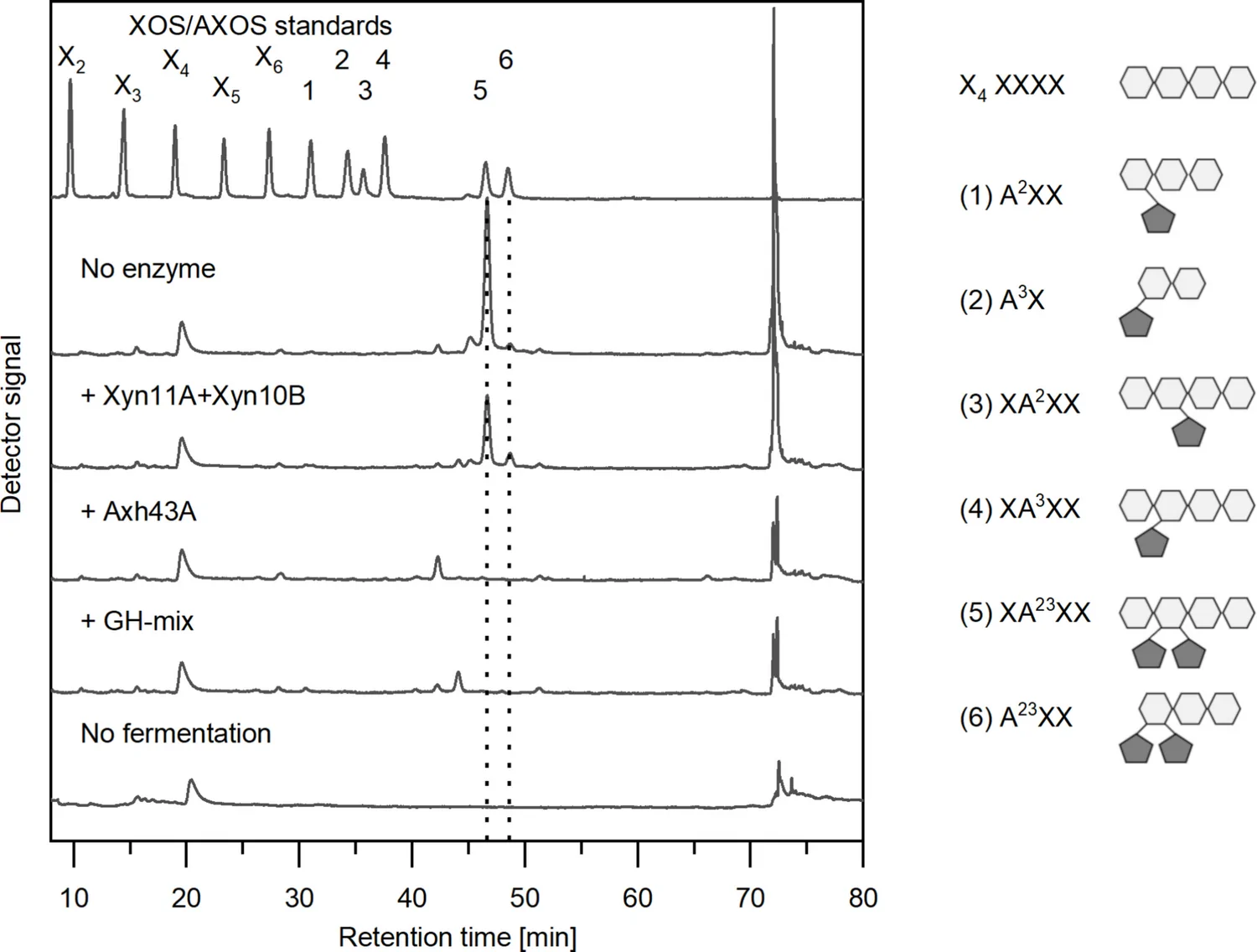
HPAEC-PAD analysis of xylooligosaccharides (XOS) and arabinoxylooligosaccharides (AXOS) in the culture supernatant after growth of *C. saccharobutylicum* RD2-A for 48 h in CGMr6 medium containing 0.25% (w/v) soluble wheat AXN. The enzymes indicated (at final concentrations of 1.5 μg/mL for Axh43A, 5 μg/mL for Xyn11A, and 5 μg/mL for Xyn10B) or a cocktail of all enzymes (GH-Mix) were added shortly before inoculation with *C. saccharobutylicum* RD2-A. The “No enzyme” chromatogram corresponds to a culture without supplementation with *T. stercorarium* DSM8532^T^ enzymes. The “No fermentation” control was not inoculated. The structures of xylotetraose and the AXOS standards (5 mg/L each) are depicted on the right side, with chains of β−1,4-linked D-xylose moieties drawn as white hexagon symbols and *O-*2- and *O-*3-linked L-arabinose substituents drawn as pentagon symbols in gray color

To understand the bottlenecks of AXN degradation by *C. saccharobutylicum* more precisely, characterization of the oligosaccharides remaining in the culture broth with and without enzyme addition was performed with HPAEC-PAD analysis (Fig. 4 and Supplemental Fig. S8). In the first phase of fermentation, many XOS and AXOS accumulated temporarily (X_2_, X_3_, X_4_, X_5_, X_6_, A^2^XX, A^3^X, XA^23^XX, A^23^XX). All XOS and AXOS were utilized by the strain, except for XA^23^XX. This hetero-oligosaccharide steadily accumulated and was the only dominant AXOS in the culture supernatant after 48 h of growth on AXN, which indicates the inability of *C. saccharobutylicum* to take up or extracellularly hydrolyse XA^23^XX. Interestingly, another double-arabinofuranosylated oligosaccharide A^23^XX could be detected transiently at 6–10 h of fermentation but was no longer found at the end of the fermentation (see Supplemental Fig. S8), suggesting consumption by *C. saccharobutylicum.*

The addition of a mixture of endoxylanases Xyn10B and Xyn11A led to a reduction in the concentration of XA^23^XX after 48 h of fermentation. Generally, GH10 xylanases such as Xyn10B exhibit broader substrate specificity but lower specific activity compared to GH11 xylanases. Xyn10B, unlike Xyn11A, can cleave xylosidic linkages adjacent to substituted xyloses at the non-reducing end of xylooligosaccharides (Chakdar et al. 2016; McCleary et al. 2015). The conversion of XA^23^XX to A^23^XX by xylanase Xyn10B was already experimentally verified (Broeker et al. 2018). However, the limited degradation observed in our experiment suggests that the enzymatic activity was insufficient for a 48-h time frame.

Upon supplementation with the α-L-arabinofuranosidase Axh43A, XA^23^XX was no longer detectable after 48 h, indicating complete hydrolysis of this AXOS species. In addition, Axh43A significantly reduced the levels of high-molecular-weight unspecified AXOS that typically elute around 72 min in the chromatogram washing step. This supports previous observations that Axh43A acts directly on polymeric soluble wheat AXN (Broeker et al. 2018) and enables further backbone cleavage.

These findings provide strong evidence that double arabinofuranosyl substitutions on xylose residues represent a major enzymatic barrier in the degradation of AXN by *C. saccharobutylicum*. The enzyme Axh43A, a thermophilic extracellular glycoside hydrolase from *T. stercorarium* DSM 8532^T^, has been shown to remove α-L-arabinofuranosyl residues from both the *O-*2- and *O-*3-positions of mono- and di-substituted xylose units (Broeker et al. 2018). Based on its substrate specificity, Axh43A is classified as an AXH-md 2,3-type arabinofuranosidase (Vandermarliere et al. 2009; Ferré et al. 2000; Pitson et al. 1996; Poria et al. 2020).

### Heterologous expression of *T. stercorarium* Axh43A in *C. saccharobutylicum*

**Fig. 5:**
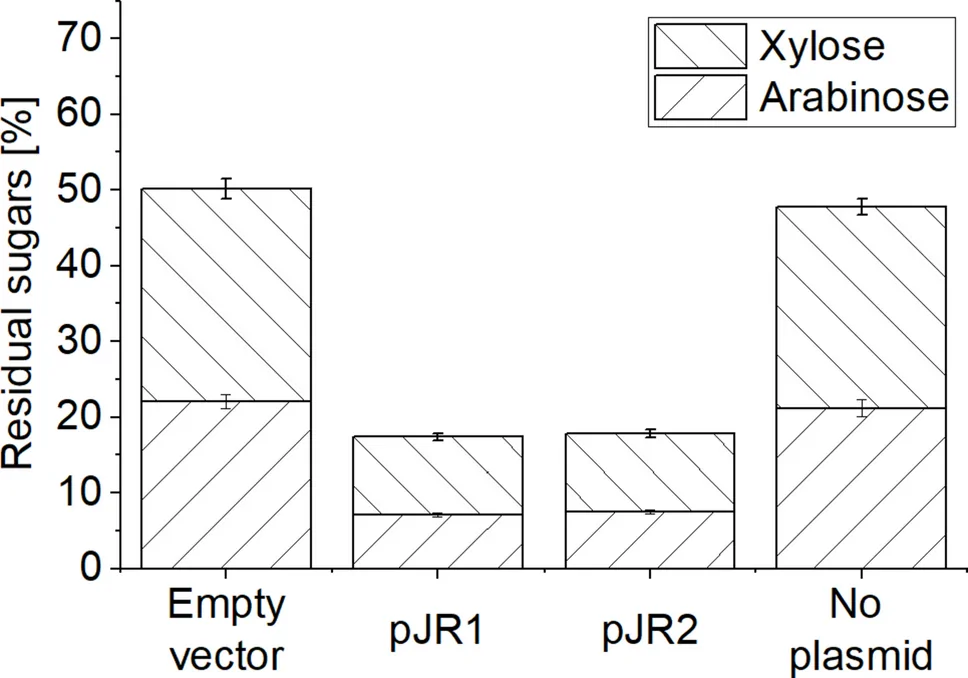
Residual sugar after 48 h fermentation of 0.25% soluble wheat AXN with *C. saccharobutylicum* RD2-A with and without plasmids in CGMr6 medium. Empty vector: pMTL83123; pJR1 contained the *T. stercorarium* DSM 8532^T^ *axh43A* ORF encoding full-length arabinofuranosidase Axh43A; pJR2 contained an ORF encoding a truncated version of *axh43A1*(∆Asn759 – Glu1221). Error bars depict standard deviations of four replicates

A strategy for a faster and more efficient substrate degradation in solvent fermentations can be an addition of enzymes (Beri et al. 2020). To reduce costs, however, heterologous expression of the necessary exoenzymes in the production strain would be desirable. To assess the possibility of expression of the gene for the *T. stercorarium* DSM 8532^T^ secretory enzyme Axh43A in *C. saccharobutylicum,* we cloned the native *axh43A* gene and a truncated version encoding the two N-terminal GH43_10 and GH43_C2 domains, but without the sequence encoding its C-terminal GH43_16 and CBM6 domain (∆N759 – E1221). In earlier experiments, the truncated version alone was still capable of cleaving doubly substituted AXOS (Baudrexl 2023). This smaller enzyme is only about 60% of the length of the original enzyme and was anticipated to perhaps be advantageous concerning expression and secretion in the heterologous host. The coding sequences for a full-length Axh43A and the truncated variant were cloned into the shuttle vector pMTL83123*.* This yielded the recombinant plasmids pJR1 and pJR2 (Supplemental Fig. S5) and allowed the heterologous expression with the native N-terminal signal peptide under the control of the P_*fdx*_ ferredoxin promoter from *C. sporogenes*. After conjugation into the restriction-negative *C. saccharobutylicum* RD2-A strain by triparental conjugation, a fermentation experiment with AXN as the substrate was carried out as before. The culture inoculated with *C. saccharobutylicum* RD2-A containing the empty plasmid (pMTL83123) reached the same level of residual sugar as the culture with *C. saccharobutylicum* RD2-A (50.2% ± 2.2 and 47.8 ± 2.2%, respectively) (Fig. 5). Strains carrying either pJR1 or pJR2 on the other hand showed improved AXN degradation and utilization (17.4 ± 0.7% and 17.8 ± 0.8% residual sugars, respectively). The effect was similar to the effect observed upon addition of purified Axh43A to the fermentation, indicating successful expression of the *T. stercorarium* DSM 8532^T^ arabinofuranosidase in *C. saccharobutylicum.*

## Discussion

Our findings demonstrate that the industrially relevant strain *C. saccharobutylicum* DSM 13864^T^ exhibits only a limited ability to utilize purified soluble AXN, a major polysaccharide component of cereal brans. Although we were able to show that the strain is natively capable of depolymerizing parts of the soluble AXN polymer into short-chain XOS (degree of polymerization 2–6) and AXOS (see Supplemental Fig. S8), its degradation capability is restricted to non-decorated or mono-arabinofuranosylated oligosaccharides. Critically, the strain lacks enzymatic activity against the *O-*2*-* and* O-*3-di-arabinofuranosylated AXOS, which accumulate (Fig. 4) and contribute to poor overall AXN conversion. By testing different enzymes from *T. stercorarium* DSM 8532^T^ for AXN degradation, we could identify Axh43A, an α-L-arabinofuranosidase capable of fully cleaving off both arabinofuranosyl sidechains, as a means to overcome this bottleneck in AXN degradation by *C. saccharobutylicum*.

The supplementation of AXN fermentations by *C. saccharobutylicum* with a mixture of either a GH11 or a GH10 xylanase (Xyn11A and Xyn10B, respectively) with Axh43A or supplementation of the fermentation with all three enzymes had almost the same effect as the addition of Axh43A alone (see Fig. 3). This result clearly supports the notion that the bottleneck for efficient AXN degradation is the missing ability to cleave off arabinose substituents from *O-*2- and *O-*3 double-arabinofuranosylated xyloses. Xylan backbone modifications are known to block or reduce the activity of endoxylanases, especially of true xylanases (GH11) which need at least a row of three unsubstituted xyloses to cleave the backbone (Chakdar et al. 2016; Broeker et al. 2018). Xylanases of the GH10 family are less sensitive, but it is known that they are also blocked by β−1,3 substitutions on the xylose at the non-reducing side of the chain (Fujimoto et al. 2004; Pell et al. 2004; Biely et al. 2013). After sidechain removal by Axh43A, native *C. saccharobutylicum* endoxylanases can continue the breakdown. The production of native endoxylanase activity in *C. saccharobutylicum* is evident from the result that roughly half of the AXN in fermentations was degraded by *C. saccharobutylicum* without enzyme addition (around 50% residual sugar content in different fermentations; see Figs. 1, 3, 5). The native endoxylanase activity of *C. saccharobutylicum* also explains the limited effect of the supplementation of heterologous xylanases Xyn11A and Xyn10B in addition to Axh43A on the extent of AXN degradation (Fig. 3).

In addition to enhancing AXN utilization by *C. saccharobutylicum*, Axh43A or other enzymes with similar cleavage characteristics may be of more general value to support AXN utilization by other non- or poor AXN-degrading bacteria. The positive effect that Axh43A has on AXN utilization by *C. saccharobutylicum* also demonstrates the critical role of accessory debranching enzymes in unlocking the fermentable sugar potential of highly decorated hemicellulose structures (Pentari et al. 2025; Sørensen et al. 2007).

To date, only a few enzymes with activity on doubly *O-2-* and *O-3-*arabinofuranosylated xyloses have been identified—specifically those belonging to the glycoside hydrolase subfamilies GH43_10 and GH43_36, as well as an enzyme purified from barley (Long et al. 2024; Ferré et al. 2000). Other α-L-arabinofuranosidases active on AXN are found in glycoside hydrolase families GH2, GH3, GH43, GH51, GH54, GH62, and GH159 (Poria et al. 2020). However, their activity is limited: Characterized enzymes from GH51, GH62, and some of GH43 act only on monosubstituted xyloses (Koutaniemi and Tenkanen 2016; Sørensen et al. 2006; Sakamoto et al. 2011); members of GH54 and some of GH43 may remove only the *O-*3-linked arabinose from double-substituted xyloses (Koutaniemi and Tenkanen 2016; Sørensen et al. 2007), and GH159 enzymes can remove *O-*2- and *O-*3-arabinofuranosyl groups from double-substituted xyloses but act only on AXOS when the substituted xylose is located directly at the non-reducing end of the xylose backbone (Baudrexl 2023). As Axh43A can fully cleave off both substitutions, it is especially interesting for further application in fermentation processes that utilize hemicellulose-rich substrates.

While purified wheat AXN served as a useful model substrate, native cereal bran contains additional glycosidic and non-glycosidic modifications. This can include acetylation, feruloylation, and p-coumaroylation, which are typically lost during mild alkaline extraction (communication from Megazyme Ltd., Bray, Ireland). Although these modifications may occur at lower abundance, they can hinder enzymatic access and downstream degradation. To further enhance biomass conversion, integration of accessory enzyme classes such as carbohydrate esterases should be considered, as previously highlighted (Mafa et al. 2021; Liu et al. 2022; Pereira et al. 2021).

Our data indicate that plasmid-based expression of Axh43A worked well in *C. saccharobutylicum* and can be used to demonstrate the usefulness of heterologous enzyme production to provide degradative functions not produced by the host bacterium itself. To improve plasmid stability during cloning in *E. coli* and to enable controlled expression in the host, the use of inducible promoters is advantageous. A promising solution for both problems could be achieved by the use of the synthetic LacO-repressed promoters (Willson et al. 2016; Mingardon et al. 2005) or the alternative sigma factor–based expression system based on *tcdR* (Omorotionmwan et al. 2023). To achieve strain stability under industrial conditions, future efforts should aim for chromosomal integration of the gene for Axh43A while simultaneously ensuring a sufficient level of expression of this important enzyme.

In summary, this work demonstrates that targeted enhancement of only one degradative enzyme activity can significantly improve AXN degradation efficiency of *C. saccharobutylicum* and potentially also other weak AXN hydrolyzing solventogenic clostridial strains. These findings provide a foundation for rational strain development and broader exploitation of lignocellulosic feedstocks in industrial fermentation processes.

## Supplementary Information

Below is the link to the electronic supplementary material.

ESM1 (DOCX 2.59 MB)

## Data Availability

The data sets used and/or analyzed during the current study are available from the corresponding author on reasonable request.
