# Supplementary material for: Improvement of arabinoxylan degradation in Clostridium saccharobutylicum DSM 13864T fermentations by heterologous glycoside hydrolase supplementation and expression
Source: Appl Microbiol Biotechnol. 2025 Dec 19;109:277. doi: 10.1007/s00253-025-13670-4 (PMC12718284; doi:10.1007/s00253-025-13670-4)
Supplement: Supplementary file 1 [file 253_2025_13670_MOESM1_ESM.docx]

**Applied Microbiology and Biotechnology**

**Supplemental material:**

**Enhancing arabinoxylan degradation in fermentations with *Clostridium*** ***saccharobutylicum* DSM 13864**

Holger Edelmann^1^, Joseph Rebel, Melanie Baudrexl^1^, Wolfgang Liebl^1^* and Armin Ehrenreich^1^*

^1^Technical University of Munich, Chair of Microbiology, Emil-Ramann-Str. 4, 85354 Freising, Germany

*Corresponding authors:

Armin Ehrenreich, orcid.org/0000-0001-9580-3620, E-mail: aehrenr@tum.de

Wolfgang Liebl, orcid.org/0000-0002-8421-2478, Phone: +49 816171-5450, E-mail: wliebl@tum.de

Figure S1. SDS-PAGE analysis of the His₆-tag affinity purification of the *T. stercorarium* DSM 8532 xylanase Xyn10B. The enzyme was heterologously expressed with a C-terminal His_6_-tag in *E. coli* BL21 (DE3) and purified by immobilized metal affinity chromatography (IMAC) as described before (Mechelke et al. 2017) using an ÄKTA pure™ chromatography system. Samples were collected at various steps of the purification process: sonicated crude lysate (S1), supernatant after centrifugation of the lysate (S2), pellet fraction (S3), flow-through during column loading (S4), and wash fraction (S5). Elution was performed in seven separate fractions (F1–F7), which were pooled and subsequently incubated at 50 °C for 15 minutes (CF). Prior to electrophoresis, all samples were mixed with 4× SDS sample buffer and heated at 95 °C for 15 minutes. Bands corresponding to the expected molecular weight of the target protein are indicated. Protein size standard: PageRuler™ (10–180 kDa)

Figure S2. SDS-PAGE analysis of the His_6_-tag affinity purification of the *T. stercorarium* DSM8532 xylanase Xyn11A. The enzyme was heterologously expressed with a C-terminal His_6_-tag in *E. coli* BL21 (DE3) and purified by immobilized metal affinity chromatography (IMAC) as described before (Mechelke et al. 2017) using an ÄKTA pure™ chromatography system. Samples were collected at various steps of the purification process: sonicated crude lysate (S1), supernatant after centrifugation of the lysate (S2), pellet fraction (S3), flow-through during column loading (S4), and wash fraction (S5). Elution was performed in seven separate fractions (F1–F7), which were pooled and subsequently incubated at 50 °C for 15 minutes (CF). Prior to electrophoresis, all samples were mixed with 4× SDS sample buffer and heated at 95 °C for 15 minutes. Bands corresponding to the expected molecular weight of the target protein are indicated. Protein size standard: PageRuler™ (10–180 kDa).

Figure S3. SDS-PAGE analysis of the His_6_-tag affinity purification of the *T. stercorarium* DSM8532 arabinofuranoidase Axh43A. The enzyme was heterologously expressed with a C-terminal His_6_-tag in *E. coli* BL21 (DE3) and purified by immobilized metal affinity chromatography (IMAC) as described before (Mechelke et al. 2017) using an ÄKTA pure™ chromatography system. Samples were collected at various steps of the purification process: sonicated crude lysate (S1), supernatant after centrifugation of the lysate (S2), pellet fraction (S3), flow-through during column loading (S4), and wash fraction (S5). Elution was performed in seven separate fractions (F1–F7), which were pooled and subsequently incubated at 50 °C for 15 minutes (CF). Prior to electrophoresis, all samples were mixed with 4× SDS sample buffer and heated at 95 °C for 15 minutes. Bands corresponding to the expected molecular weight of the target protein are indicated. Protein size standard: PageRuler™ (10–180 kDa)

Figure S4. SDS-PAGE analysis of the His_6_-tag affinity purification of the *T. stercorarium* DSM8532 glycoside hydrolase Bxl3B. The enzyme was heterologously expressed in *E. coli* BL21 (DE3) and purified using a His-tag affinity column in combination with the ÄKTA pure™ chromatography system. Samples were collected at various steps of the purification process: sonicated crude lysate (S1), supernatant after centrifugation of the lysate (S2), pellet fraction (S3), flow-through during column loading (S4), and wash fraction (S5). Elution was performed in seven separate fractions (F1–F6), which were pooled and subsequently incubated at 50 °C for 15 minutes (CF). Prior to electrophoresis, all samples were mixed with 4× SDS sample buffer and heated at 95 °C for 15 minutes. Bands corresponding to the expected molecular weight of the target protein are indicated. Protein size standard: PageRuler™ (10–180 kDa).

Figure S5. Plasmid map of expression vectors pJR1 and pJR2. Both vectors were created by inserting *axh43A* (Cst_c07100) or a truncated version *axh43A1*(ΔN759 – E1221; containing coding regions for GH43_10 and GH43_C2 domain) into the multiple cloning site of pMTL83123. Check primers are indicated by dashed arrows. (<https://BioRender.com/r74i427>)

Figure S6. PCR amplification of *axh43A* (Cst_c07100) and the truncated variant *axh43A1* (Cst_c07100, ΔN759–E1221) using Q5 polymerase. DNA fragments were amplified from *T. stercorarium* DSM 8532 genomic DNA using Q5 High-Fidelity DNA Polymerase (NEB). The expected fragment sizes were 3,726 bp for *axh43A* and 2,337 bp for *axh43A1*. A negative control (NC) was included. PCR products were analyzed by agarose gel electrophoresis. DNA size marker: GeneRuler 1 kb DNA Ladder.

Figure S7. Restriction analysis of plasmid constructs pCAN3_JR1 and pCAN3_JR2. Plasmids were assembled via Gibson Assembly and introduced into *E. coli* DH10β. Following overnight cultivation, plasmids were extracted and digested with *EcoRI*. The expected fragment sizes were as follows: pCAN3_JR1 – 3,962 bp, 2592 bp, and 1,515 bp; pCAN3_JR2 – 3,962 bp and 2,718 bp; and the original vector pCAN3 (positive control, PC) – 3,475 bp and 1,001 bp. A negative control (NC) was included. Expected DNA fragments observed in the agarose gel are indicated. Three clones containing pCAN3_JR1 and two clones containing pCAN3_JR2 were analyzed by agarose gel electrophoresis. DNA size marker: GeneRuler 1 kb DNA Ladder.

Figure S8. Analysis of oligosaccharides in the time course of fermentation of *C. saccharobutylicum* wheat arabinoxylan (0.25%, w/v) in CGMr6 medium. Samples were taken at 6, 10, 24 and 48 hours and applied in 1:10 dilution to separation with HPAEC-PAD. AXOS-Standards were measured at a concentration of 5 mg/L.
